# The rhizosphere microbial community in a multiple parallel mineralization system suppresses the pathogenic fungus *Fusarium oxysporum*

**DOI:** 10.1002/mbo3.140

**Published:** 2013-11-08

**Authors:** Kazuki Fujiwara, Yuichiro Iida, Takashi Iwai, Chihiro Aoyama, Ryuya Inukai, Akinori Ando, Jun Ogawa, Jun Ohnishi, Fumihiro Terami, Masao Takano, Makoto Shinohara

**Affiliations:** 1 National Institute of Vegetable and Tea Science, National Agriculture and food Research OrganizationTsu, Mie, 514-2392, Japan; 2 Graduate School of Environmental Studies, Nagoya UniversityChikusa, Nagoya, Aichi, 464-8601, Japan; 3 Division of Applied Life Sciences, Graduate School of Agriculture, Kyoto UniversityKitashirakawa-oiwakecho, Sakyo-ku, Kyoto, 606-8502, Japan; 4 Research Unit for Physiological Chemistry, Kyoto UniversityKitashirakawa-oiwakecho, Sakyo-ku, Kyoto, 606-8502, Japan

**Keywords:** *Fusarium oxysporum*, multiple parallel mineralization, rhizosphere biofilm, soil-borne disease suppression.

## Abstract

The rhizosphere microbial community in a hydroponics system with multiple parallel mineralization (MPM) can potentially suppress root-borne diseases. This study focused on revealing the biological nature of the suppression against Fusarium wilt disease, which is caused by the fungus *Fusarium oxysporum*, and describing the factors that may influence the fungal pathogen in the MPM system. We demonstrated that the rhizosphere microbiota that developed in the MPM system could suppress Fusarium wilt disease under *in vitro* and greenhouse conditions. The microbiological characteristics of the MPM system were able to control the population dynamics of *F. oxysporum*, but did not eradicate the fungal pathogen. The roles of the microbiological agents underlying the disease suppression and the magnitude of the disease suppression in the MPM system appear to depend on the microbial density. *F. oxysporum* that survived in the MPM system formed chlamydospores when exposed to the rhizosphere microbiota. These results suggest that the microbiota suppresses proliferation of *F. oxysporum* by controlling the pathogen's morphogenesis and by developing an ecosystem that permits coexistence with *F. oxysporum*.

## Introduction

A soil-free cultivation system is generally believed to require the elimination of microbial and organic contaminants from the nutrient solution, whether the system uses open or closed circulation (Garland and Mackoqiak 1990; Stanghellini and Rasmussen 1994; Stanghellini et al. 1996; Garland et al. 1997; Koohakana et al. 2004; Ehret et al. 2005; Lee et al. 2006). In contrast, multiple parallel mineralization (MPM) is a novel form of hydroponics system in which microbial activity and the presence of organic nutrients in the solution lead to the development of plant–microbe ecosystems in the rhizosphere (Shinohara et al. 2011). The rhizosphere microbiota that develops in an MPM system is responsible for two reproducible functions that are necessary for sustainable plant growth: high nutrient production efficiency and the control of root diseases. The former trait results from microbial mineralization of the organic fertilizer used in the hydroponics solution, which promotes the transformation of organic nitrogen into nitrate nitrogen in the hydroponics solution as a result of two sequential microbial processes: ammonification and nitrification. To achieve this process, it is necessary to culture soil microorganisms in the hydroponics solution and develop a microbial community that is capable of mineralizing organic fertilizer into nitrate ions. This approach recreates a microbial environment that promotes coexistence of the plants and microbes in a hydroponics system in a manner similar to that which occurs in soils (Shinohara et al. 2011).

The second trait means that the MPM system has the potential to control root-borne diseases as a result of the actions of the microbiota community that develops in the rhizosphere. We have previously explored the ability of this system to suppress the bacterial wilt disease caused by *Ralstonia solanacearum*, a plurivorous phytopathogenic bacterium (Fujiwara et al. 2012). Our examination of this suppression demonstrated that the MPM system could suppress this bacterial wilt disease. We have also observed that other root-borne diseases that often damage several plant species in inorganic hydroponics systems, including lettuce, komatsuna, rice, cucumber, and pepper, did not occur in the MPM system, allowing cultivation of the plants without requiring fungicides or other antibiotics before they could be harvested (Shinohara 2006; Shinohara et al. 2011; M. Shinohara and K. Fujiwara, unpubl. data). These results revealed the importance of the microbiota that colonizes the plant rhizosphere in the suppression of root-borne diseases.

Our interest in the suppression of root diseases obtained in an MPM system led us to explore the biological nature of the disease suppression and describe the possible factors influencing root-borne pathogens in this system. Previously, the MPM system showed an ability to suppress bacterial wilt (Fujiwara et al. 2012), but the potential for controlling fungal root diseases had not yet been examined. In this study, we used the fungal root pathogen *Fusarium oxysporum*, which is an important phytopathogenic fungus for many crops (Di Pietro et al. 2003). We focused on investigating the microbiological factors that underlie the suppression of *F. oxysporum* f. sp. *lactucae*, which causes root rot and wilting of commercial lettuce plants and has become a significant problem in Japan (Fujinaga et al. 2003, 2005). Identifying the factors responsible for the success of this approach will improve our understanding of microbial contributions to the suppression of root-borne diseases and provide insights into previously unknown biological phenomena that occur in the plant rhizosphere.

## Experimental Procedures

### Hydroponics solution

The inorganic nutrient solution was prepared using inorganic fertilizers (4.8 g of Otsuka House No. 1 and 2.7 g of Otsuka House No. 2; Otsuka Chemical, Osaka, Japan) to supply 200 mg L^−1^ of nitrate ions. The MPM solution, which is a hydroponics solution used in the MPM system, was prepared by a procedure that we have called “multiple parallel mineralization” (Shinohara et al. 2011), which is described in more detail in the Fig S1–S5, Table S1, and Data S1. This procedure aimed at culturing a community of microorganisms that would be capable of mineralizing organic nitrogen into nitrate nitrogen. Briefly, we prepared 15 L of water containing 150 g of nursery soil as a microbial inoculum (Nae-ichiban; Sumirin Agro-Products, Aichi, Japan). We then added 4 g of corn steep liquor (CSL; Nature Aid; Sakata Seed, Yokohama, Japan) containing 120 mg of organic N daily for 7 days, followed by adding 150 g of oyster shell lime (Ryujyoseruka; Urabe Sangyo, Hiroshima, Japan) to the water as a supplement to provide micronutrients. The water was aerated with an aeration pump and held at room temperature for more than 2 weeks until the nitrate concentration reached at least 200 mg L^−1^ and stabilized. We regarded this stage as the point when mineralization of the CSL was complete. We used MPM solution that contained 200 mg L^−1^ of nitrate ions generated through this procedure as the MPM hydroponics solution.

### Microscopic observations

Tomato (*Solanum lycopersicum* L. cv. Ponderosa) seeds were sown in vermiculite and grown in a greenhouse (about 16 h of light and a temperature range of 10–30°C). At 2 weeks after seeding, four seedlings were transplanted into MPM solution, and cultivated in a greenhouse. At 4 days after transplanting, the roots were observed with a light microscope (BX50; Olympus, Tokyo, Japan) and a digital microscope (VHX-1000 with VHX-D500/D510 lens; Keyence, Osaka, Japan). For the scanning electron microscope, we fixed 5-mm sections of the roots twice for 2 h in 2% (v/v) glutaraldehyde at room temperature and dehydrated the sections through a graded ethanol series (20–100%), followed by immersion in 100% acetone for complete drying. Samples were coated with a thin gold layer using a JEE-400 vacuum evaporator (JEOL, Tokyo, Japan) and observed using a JSM-5800 scanning electron microscope (JEOL).

### Polymerase chain reaction–denaturing gradient gel electrophoresis and data analysis

Rice (*Oryza sativa* L. *japonica* cv. Koshihikari), komatsuna (*Brassica rapa* L. var*. perviridis* cv. Komatsuna), and tomato (*S. lycopersicum* L. cv. Ponderosa) seeds were sown in vermiculite soil and grown in a greenhouse (about 16 h of light and a temperature range 10–30°C). At 2 weeks after seeding, 16 seedlings of each species were transplanted into MPM solution. Seedlings were then cultivated in a greenhouse.

Individual plant roots (*n* = 1 per plant) were harvested from two plants at 1, 2, 3, 7, and 14 days after transplanting. The root samples were stored in 15-mL tubes containing sterilized water. After the roots were gently washed to remove any microbes or biofilms, the water was centrifuged at 10,000*g* for 5 min, and the resulting bacterial pellet was used for the denaturing gradient gel electrophoresis (DGGE) analysis.

Bacterial DNA extraction was conducted using the standard procedure (Sambrook and Russell 2001). The DNA fragments extracted from the bacterial samples were amplified using the GC-341f and GC-534r primer pair (Muyzer et al. 1993) (Table S1). Electrophoresis was performed with a Dcode DGGE complete system (Bio-Rad, Hercules, CA), using 8% polyacrylamide gel for the polymerase chain reaction (PCR) products. We used 12 h at 100 V in a linear 25–65% denaturant gradient for the electrophoresis conditions. DGGE bands were excised and reamplified using the same primers. The reamplified PCR products underwent DGGE, and the DGGE procedure was repeated at least three times, allowing for detection of a single DGGE band that represents a single bacterial species.

DNA sequences were determined using the BigDye Terminator v3.1 Cycle Sequencing Kit (Applied Biosystems, Foster City, CA) and an ABI 3130x1 Genetic Analyzer System (Applied Biosystems) using the standard methods (Sambrook and Russell 2001). The sequences have been deposited in the DDBJ/EMBL/GenBank databases under accession numbers AB605770 to AB610410. The sequences were compared with the NCBI DNA database sequences using the BLAST software (Altschul et al. 1997).

### Fungal strains and culture conditions

We used *F. oxysporum* f. sp. *lactucae* strain H111; its red fluorescent protein (dsRed; Takara Bio, Shiga, Japan) expression transformant, H111-dsRed; and *F. oxysporum* f. sp. *radicis-lycopersici* strain LS89-1-1 (Yamauchi et al. 2005). We constructed the dsRed expression vector pTEFRFP, which is driven by the *Aureobasidium pullulans* TEF promoter of pTEFEGFP (Vanden Wymelenberg et al. 1997), using the primers Ptef-RFP, Tgla-RFP, RFP-F, and RFP-R (Table S1) and the In-Fusion dry-down PCR cloning kit (Clontech, Mountain View, CA). Protoplast preparation and the cotransformation of *F. oxysporum* strain H111 were performed as previously described (Iida et al. 2008). Transformants carrying *hph* were selected on regeneration medium containing hygromycin B (Wako Pure Chemicals, Osaka, Japan) at 100 *μ*g mL^−1^ (Iida et al. 2008).

Strains were routinely maintained on potato dextrose agar (PDA; Difco, Detroit, MI) at 4°C. PDA agar blocks (3 mm in diameter) carrying mycelia were inoculated into 100 mL of potato dextrose broth (PDB; Difco) in a flask and cultured on an orbital shaker (120 rpm) at 25°C for 4 days in the dark. Subsequently, the culture was filtered through four layers of sterile gauze, and the filtrate, which contained bud cells, was centrifuged at 10,000*g* for 5 min. The cell pellet was resuspended in 50 mL of sterile distilled water and centrifuged three times at the same settings. The cell pellet was resuspended with sterile distilled water, and the final concentration was adjusted to 1 × 10^4^ cells mL^−1^ of sterile distilled water for use as inoculum.

Growth inhibition of *F. oxysporum* was tested in response to bacterial isolates or biofilm bacteria from the MPM solution. For bacterial isolates, bacterial collection was generated from the rhizosphere biofilms in the MPM solution. The MPM solution was spread plated in 1/10 nutrient broth medium (0.3 g beef extract, 0.5 g peptone, and 15 g agar), lysogeny broth medium without NaCl (10 g tryptone, 5 g yeast extract, and 20 g L^−1^ agar), CSL medium (0.1 g CSL, 0.2 g CaCO_3_, and 15.0 g L^−1^ agar, pH 6.5), or PDA medium, and plates were incubated at 25°C for 5 days. Bacterial colonies from each plate were picked up and transferred into new plates and incubated again. This was repeated twice, resulting in a collection of 204 isolates. Each agar block (3 mm in diameter) carrying a single bacterial species was placed on the abovementioned plates with strain H111. Plates were incubated in the dark at 25°C for 5 days. For biofilm bacteria, assays were performed on PDA medium containing hygromycin B at 100 μg mL^−1^. Five millimeter of a filter paper was soaked in biofilm collected from the MPM solution and placed on a plate with strain H111-dsRed. Plates were incubated in the dark at 25°C for 5 days.

### Inoculation test

Boston lettuce (*Lactuca sativa* L. cv. Saradana) and tomato (*S. lycopersicum* L. cv. Ponderosa) cultured in one of two hydroponics systems were used for the inoculation test. Seeds were sown in vermiculite soils. At 2 weeks after seeding, four seedlings of each species were transplanted into separate 1.5-L plastic pots filled with MPM solution or the abovementioned inorganic hydroponics solution. At 4 days after transplanting, each of the seedlings was inoculated with a bud cell suspension of *F. oxysporum* (1 × 10^4^ cells mL^−1^). There were three replicates (three pots with four plants each) for each plant species–solution combination. All the experiments were performed at 32°C with a 12-h photoperiod. Disease symptoms were primarily observed starting 6 days after inoculation.

Samples of the hydroponics solution were directly collected and incubated at 25°C for 3 days on Komada's selective medium (1.0 g Na_2_B_4_O_7_·10H_2_O, 1.0 g K_2_HPO_4_, 0.5 g KCl, 0.5 g MgSO_4_·7H_2_O, 0.01 g Fe-Na-ethylenediaminetetraacetic acid, 2.0 g l-asparagine, 20.0 g d-galactose, 15.0 g agar, 1.0 g pentachloronitrobenzene, 0.5 g sodium cholate, and 0.3 g L^−1^ streptomycin sulfate) supplemented with hygromycin B at 100 μg mL^−1^ (Komada 1975). Individual plant roots were harvested from each of the three plants and suspended in 40 mL of sterile distilled water. The root samples were shaken vigorously for 1 min on a vortex mixer to remove biofilm from the root surface.

### A test for growth suppression and morphological observation of F. oxysporum

*Fusarium oxysporum* strain H111-dsRed was added to a test tube filled with 5 mL of the MPM solution at a final density of 1 × 10^2^ cells mL^−1^. To test whether the suppression resulted from filterable or autoclavable agents in the solution, the MPM solution was filtered through a 0.2-*μ*m membrane (Millipore, Billerica, MA) or autoclaved for 20 min at 121°C. The inorganic solution was prepared and inoculated similarly. The inoculated solutions were cultured in the dark at 25°C on a shaker (170 rpm). From each replicate, solution samples were collected 3, 7, and 14 days after the experiment started and incubated at 25°C for 3 days on Komada's selective medium supplemented with hygromycin B at 100 μg mL^−1^.

A heat-treatment experiment was performed with the MPM solution, which was pasteurized at 40–80°C for 30 min. The inoculated solutions were then cultured in the dark at 25°C on a shaker (170 rpm). From each replicate, solution samples were collected 7 days after the experiment started and were incubated on Komada's selective medium. All the in vitro experiments used three replicates.

To test the ability to transfer the MPM solution's suppressive properties, the solution was centrifuged at 10,000*g* for 5 min to collect a microbial pellet. Approximately, 1.7 (±0.2) mg mL^−1^ of microbes were collected from the microbial pellet, which showed that ∼3.8 × 10^9^ colony forming units (CFU) g^−1^ or 1.8 × 10^13^ CFU g^−1^ of culturable bacteria were detected in the CSL medium and the 1/10 nutrient broth medium, respectively. The microbial pellet was then resuspended in sterile distilled water and centrifuged again at the same rate and duration. This washing step was repeated three times. The resulting microbial solution was diluted to 50, 20, 10, 5, and 2% of the original concentration by adding sterile distilled water. The diluted solutions were centrifuged, and the resulting microbial pellets were resuspended in 5 mL of the inorganic hydroponics solution.

In our morphogenetic study, *F. oxysporum* strain H111-dsRed was added to a test tube filled with 5 mL of the MPM solution or the inorganic hydroponics solution at a final density of 1 × 10^4^ cells mL^−1^. The inoculated solutions were cultured in the dark at 25°C for 7 days on a shaker (170 rpm), with three replicates. At 7 days after the inoculation, fungal cells were observed using a fluorescence microscope (BX50) equipped with a U-MWIG filter (Olympus). To assess *F. oxysporum* growth, more than 200 cells were observed from each replicate. The total number of microconidia, germinated microconidia (<20 *μ*m), elongated hyphae (>20 *μ*m), macroconidia, and chlamydospores was counted.

## Results

### The rhizosphere microbial community

**Figure 1:**
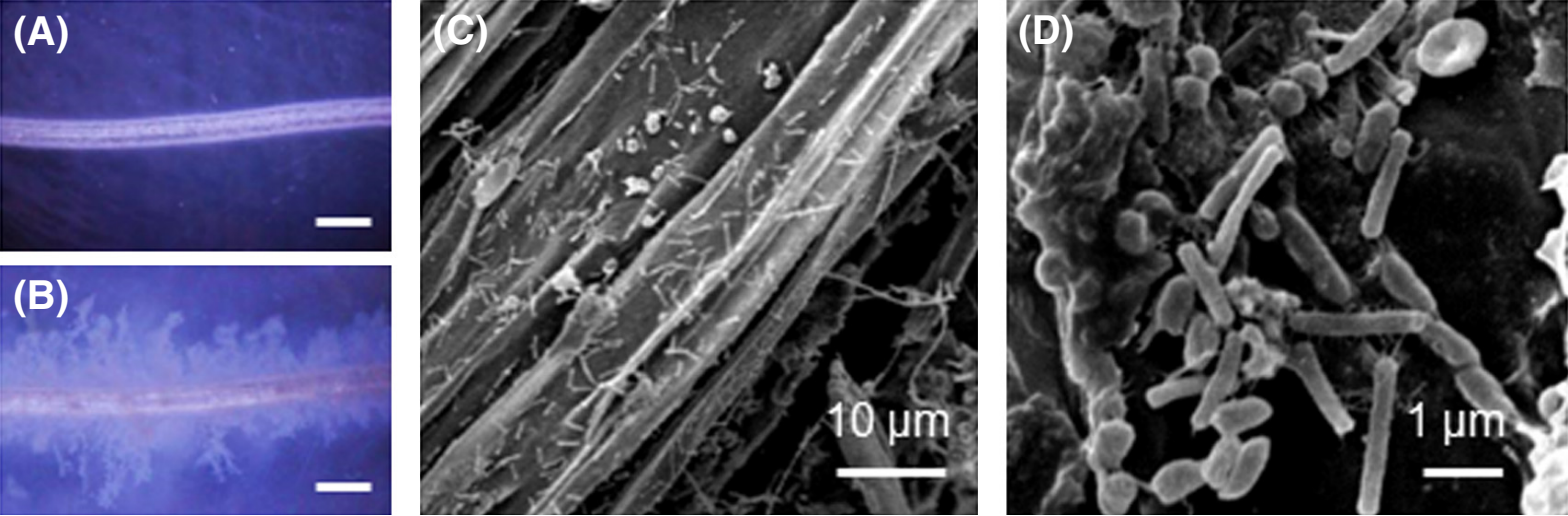
Biofilms adhered to the root surface in the multiple parallel mineralization (MPM) system but not in the inorganic hydroponics system. Tomato roots were grown in (A) a conventional inorganic system and (B) in the MPM system for 4 days after transplanting and were observed with a light microscope. Bars for A and B represent 1 mm. Seedlings were cultivated in a greenhouse at a constant day and night temperature of 30°C. (C, D) Rhizosphere microbes inhabiting the root surface in the MPM system under a scanning electron microscope. See Figure S1 for additional images.

We observed rhizosphere biofilms developing on the roots under a microscope and a scanning electron microscope. Root hairs developed, and a biofilm covered the root hairs in the MPM system, whereas neither root hairs nor biofilm was observed in the conventional inorganic hydroponics (Fig. 1A, B). Bacteria detected at ∼10^5^ CFU mL^−1^ in the inorganic hydroponics solution could not be observed under the microscope. Microbial colonization was obvious both on root hairs and in zones where mature main and lateral roots were present (Figs. 1C, S1). Several microbes with different morphologies were detected on the root surface, and some microbes adhered tightly to the root surface and to other microbial cells (Fig. 1D). In short, rhizosphere microorganisms indigenous to the MPM solution colonized the roots and were associated with biofilm formation on the rhizoplane.

**Figure 2:**
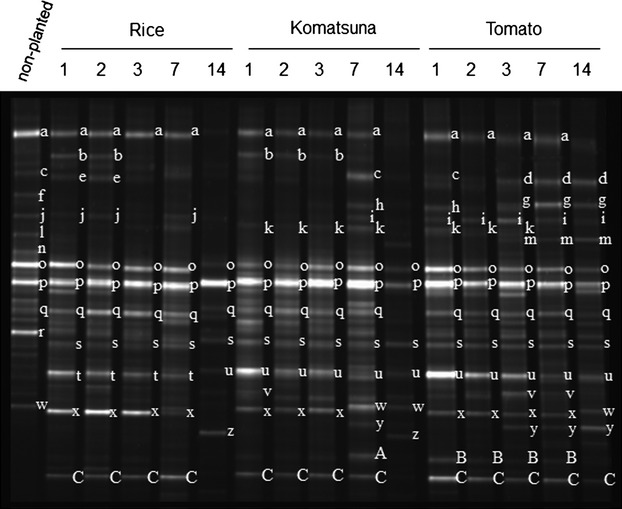
Denaturing gradient gel electrophoresis (DGGE) banding profiles for the bacterial 16S rDNA genes extracted from the rhizosphere biofilms that developed in the multiple parallel mineralization (MPM) system. Plants were grown in the MPM system for 2 weeks in a greenhouse. Lane numbers (1, 2, 3, 7, and 14) correspond to the number of days after transplanting. The root samples were washed in sterilized water, and the resulting bacterial solution was collected by centrifugation. The bacterial pellets were used for the DGGE analysis. A control sample (“non-planted”) represents a biofilm that developed in the MPM solution before cultivation of the plants. Bands with the same mobility in the DGGE gel were marked with the same letter (a–z and A–C) and were excised for sequencing.

Classification of the rhizosphere bacteria in the MPM system was carried out by means of PCR–DGGE analysis, which detected 174 bands from a series of biofilms that developed before and after plant cultivation (Fig. 2). We successfully identified 11 bands for organisms extracted from the MPM solution before plant cultivation, 45 bands from rice, 51 bands from komatsuna, and 67 bands from tomato by comparative analysis of their 16S rDNA genes (Table 1). Rhizosphere bacteria present in the DGGE profiles belonged to bacterial phylogenetic groups known to exist in the soil. The bacteria were affiliated with 17 phylogenetic groups (Table 1). The DGGE profiles were assigned to phylogenetic groups of four bacterial phyla (Actinobacteria, Bacteroidetes, Firmicutes, and Proteobacteria) on the basis of reproducible phylogenetic affiliations (Fig. S2). The 174 bands were assumed to be operational taxonomic units (OTUs) and assigned to bacterial phyla. The overall distribution of the identified bacterial phyla was ∼20% for Actinobacteria, Bacteroidetes, and Proteobacteria, respectively, versus ∼40% for Firmicutes.

**Table 1:** Phylogenetic affiliations of the bacterial 16S rDNA gene sequences corresponding to the prominent DGGE bands retrieved from the rhizosphere biofilms.

DGGE band^1^	Microorganisms	Phylogenetic affiliation	Accession number	Identity (%)^2^	Alignment
a	*Lysinibacillus* sp.	Bacillaceae	AB605770	100	194/194
b	*Variovorax paradoxus*	Burkholderiales	AB608026	99.5	193/194
c	Uncultured sphingobacteria bacterium	Sphingobacteriales	AB610409	96.8	183/189
d	Uncultured deltaproteobacterium	Deltaproteobacteria	AB608046	97.9	190/194
e	Uncultured bacterium	Deltaproteobacteria	AB608031	96.9	188/194
f	*Niastella* sp.	Chitinophagaceae	AB607235	98.4	186/189
g	Uncultured bacteroidetes bacterium	Chitinophagaceae	AB608045	98.4	186/189
h	Uncultured bacteroidetes bacterium	Bacteroidetes	AB608040	98.4	186/189
i	Uncultured Sphingobacteriales bacterium	Sphingobacteriales	AB608044	100	189/189
j	*Comamonas* sp.	Comamonadaceae	AB607236	99.0	192/194
k	Uncultured deltaproteobacterium	Deltaproteobacteria	AB608041	94.3	183/194
l	*Olivibacter terrae*	Sphingobacteriaceae	AB605771	97.4	184/194
m	Uncultured *Comamonas* sp.	Comamonadaceae	AB608047	100	194/194
n	*Bacillus niabensis*	*Bacillus*	AB607230	99.5	193/194
o	*Bacillus* sp.	*Bacillus*	AB607231	100	195/195
p	*Bacillus* sp.	*Bacillus*	AB607232	100	194/194
q	*Bacillus horneckiae*	*Bacillus*	AB608027	100	194/194
r	*Bacillus* sp.	*Bacillus*	AB607233	100	194/194
s	*Rhizobium* sp.	*Rhizobium*	AB608042	100	169/169
t	*Dyadobacter* sp.	Dyadobacter	AB608028	98.9	187/189
u	*Bacillus* sp.	Bacillaceae	AB608035	97.4	190/194
v	Uncultured bacteroidetes bacterium	Bacteroidetes	AB608043	95.2	180/189
w	Uncultured Cytophagales bacterium	Cytophagales	AB607234	99.5	185/186
x	Uncultured *Microbacterium* sp.	Microbacteriaceae	AB608029	100	174/174
y	Uncultured bacterium	Bacteroidetes	AB608048	99.5	188/189
z	*Chelativorans* sp.	Rhizobiales	AB608033	100	169/169
A	Uncultured actinobacterium	Actinomycetales	AB608039	94.8	165/174
B	Uncultured actinobacterium	Thermomonosporaceae	AB610410	99.4	167/168
C	*Gordonia* sp.	*Gordonia*	AB608030	100	174/174

1 DGGE bands used for the sequences were excised from the DGGE banding profiles (Fig. 2).

2 Percent similarity of the partial 16S rDNA coding sequences to the sequences of their closest bacterial relatives available based on a BLAST search in the NCBI nucleotide sequence database. DGGE, denaturing gradient gel electrophoresis.

The diversity of the bacterial community structure based on the OTU compositions in each bacterial phylum was estimated using a unweighted pair group method with arithmetic mean (UPGMA) dendrogram to identify the biofilm samples with the highest similarity in their rhizosphere microbial community structures. The OTU compositions showed that the bacterial community before plant growth (nonplanted) was clustered apart from the community around each plant species (Fig. S3). The bacterial community associated with the early growth stages (within 7 days after transplanting) and the later growth stages (14 days after transplanting) was clustered separately (*F*_(2,13)_ = 5.25, *P* < 0.005, *R*^2^ = 0.45), with one exception; the community obtained from tomato after 14 days was clustered with the earlier stages (*P* > 0.05) (Fig. S3).

In terms of the abundance of several bacterial taxa (Fig. 2, Table 1), three band profiles that represented *Bacillus* (o, p, and q) were the dominant band patterns both before and after plant cultivation. In addition, five band profiles that represented Bacillaceae (u, in komatsuna and tomato), *Gordonia* (C, in all three species), Sphingobacteriales (i, in tomato), *Bacillus* (q, in all three species), and *Rhizobium* (s, in all three species) remained constant during plant cultivation (Fig. 2). In contrast, bacteria in the Chitinophagaceae (f), Sphingobacteriaceae (l), and *Bacillus* (n and r) were observed only before plant cultivation (Fig. 2). The following bacteria from the rhizosphere of the MPM system (Table 1) were among the bacterial taxa that are known as biocontrol agents (Raaijmakers et al. 2002; Clardy et al. 2006; Borneman and Becker 2007): *Bacillus* (n, o, p, q, r, and u) and Actinomycetales (A). Altogether, the population-based approach using DGGE analysis indicated that the microbiota inhabiting the MPM system involves a core bacterial group that includes several dominant bacteria that are associated with disease suppression (Mendes et al. 2011).

### Suppression of fungal disease in the MPM system

The fungal pathogen *F. oxysporum* f. sp. *lactucae* causes root rot and wilting of commercial lettuce plants in Japan (Fujinaga et al. 2003, 2005). The virulence of the fungal disease makes it a widespread problem in hydroponics systems based on an inorganic nutrient solution. We used transformants that constitutively express the dsRed and hygromycin B resistance genes to detect the inoculated *F. oxysporum* strain. The expression vector pTEFRFP was introduced into *F. oxysporum* f. sp. *lactucae* strain H111 by cotransformation with the plasmid pSH75, which confers hygromycin B resistance. We observed the mycelia of transformants grown on PDA by means of fluorescence microscopy. Of the 30 transformants from H111, 12 expressed dsRed. We used these H111-dsRed transformants in the subsequent experiments.

**Figure 3:**
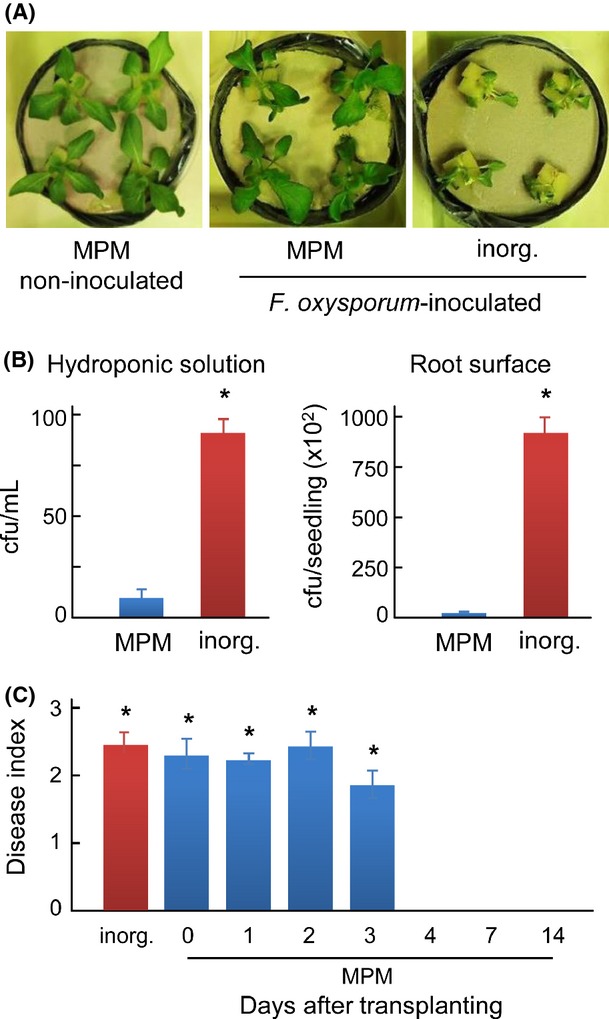
Development of suppression of Fusarium wilt disease in the multiple parallel mineralization (MPM) system. (A) Boston lettuce plants were inoculated with *Fusarium oxysporum* f. sp. *lactucae* strain H111-dsRed (1 × 10^4^ cells mL^−1^) 4 days after transplanting. (B) Bacterial samples were collected from the inorganic (inorg.) and MPM solutions and root surfaces. The strain H111-dsRed in the bacterial samples was detected using Komada's selective medium supplemented with hygromycin B. Bars labeled with a star were significantly different (*P* < 0.05, to a Student's *t*-test). (C) Boston lettuce plants grown in the MPM system were inoculated with the strain H111-dsRed (1 × 10^4^ cells mL^−1^) 0–14 days after transplanting, and then the disease severity was monitored after the inoculation. The disease severity was determined on a scale of 0–3, in which 0 indicates no disease, 1 indicates wilting leaves, 2 indicates a wilting plant, and 3 indicates a dead plant, and symptoms were recorded until 10 days after inoculation. The disease rating represents the mean score from 12 seedlings in each treatment replicate. Disease severity in the inorganic hydroponics system (inorg.) is shown as a control. Bars labeled with a star were not significantly different from the inorganic solution (*P* < 0.05, Tukey–Kramer multiple-comparison test).

When Boston lettuce seedlings were inoculated with *F. oxysporum* f. sp. *lactucae* strain H111 under greenhouse conditions, the MPM system provided remarkable suppression of *F. oxysporum*, whereas conventional inorganic hydroponics showed a high disease incidence and severe damage (Fig. S4A). When strain H111-dsRed was inoculated into the hydroponics solutions of the lettuce under growth chamber conditions, typical symptoms were observed for plants grown in the conventional inorganic hydroponics system, but not in the MPM system 4 days after transplanting (Fig. 3A). Infected seedlings began to appear in the inorganic hydroponics system 6 days after inoculation and wilted shortly after disease symptoms had appeared on the leaves (Table S2). In contrast, no disease symptoms were observed in the MPM system (Fig. 3A). The suppressive effect was sustained until harvesting. The suppression of *F. oxysporum* f. sp. *radicis-lycopersici* was also observed in tomato plants. Strain LS89-1-1 (1 × 10^4^ cells mL^−1^) was inoculated on the tomato plants 4 days after transplanting, and all plants in the inorganic hydroponics system (but not the MPM system) died or became severely stunted within 10 days after inoculation (Fig. S4B, Table S2). These results demonstrated that the MPM system strongly suppressed the Fusarium wilt disease even under greenhouse conditions.

*Fusarium oxysporum* survived in the rhizosphere microbiota despite the disease suppression. We detected abundant *F. oxysporum* in both infected plant root surfaces and the hydroponics solution of the inorganic system (Fig. 3B). However, surprisingly, we also detected the fungal pathogen (although at lower levels) in the rhizosphere biofilms that formed on the root surface and in the hydroponics solution in the MPM system, although seedlings in the MPM system showed no disease symptoms (Fig. 3B). We reisolated the strains of *F. oxysporum* from the MPM solution and confirmed their strong pathogenicity to lettuce plants (data not shown). These results showed that the development of Fusarium wilt disease was strongly suppressed in the MPM system, even though the pathogen was detected in the rhizosphere. They also indicated that the suppression of Fusarium wilt disease resulted from control of fungal activity rather than from destruction of the pathogen.

To clarify the effects of transplanting on disease control, we performed an inoculation test under growth chamber conditions. Plants were inoculated with strain H111 from 0 to 14 days after transplanting in the MPM system. Interestingly, the disease suppression developed only 4 days after transplanting (Fig. 3C). These results showed that the MPM system suppressed the development of root diseases caused by both bacterial (Fujiwara et al. 2012) and fungal pathogens if transplanted seedlings had at least 3 days to become established in the system before pathogen introduction.

### Microbiological factors responsible for disease suppression in the MPM solution

We explored the biotic and abiotic factors that contributed to the suppression of *F. oxysporum* activity in the MPM solution. Initially, we used culture-based approaches to identify antagonists against *F. oxysporum*. We assayed the ability of bacterial isolates from the rhizosphere biofilm to reduce *F. oxysporum* growth in vitro. To do so, we collected 204 isolates from the MPM solution, but none of the isolates suppressed strains H111 and LS89-1-1 on several media (data not shown).

**Figure 4:**
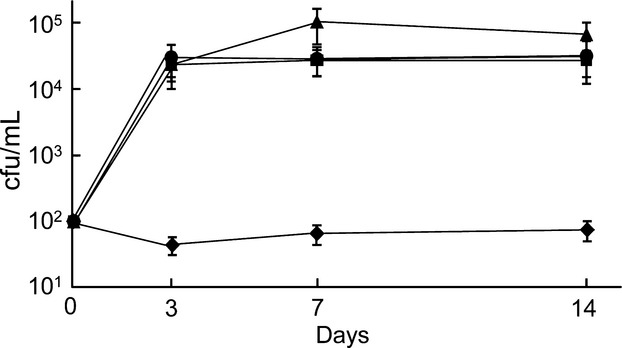
The suppression of *Fusarium oxysporum* by the multiple parallel mineralization (MPM) solution. The factors that suppressed *F. oxysporum* growth in the MPM system were evaluated by sterilization of the solution by filtration and autoclaving. The inoculated solutions were cultured at 25°C for 14 days, and were examined using Komada's selective medium supplemented with hygromycin B. *Fusarium oxysporum* f. sp. *lactucae* strain H111-dsRed was added to the MPM (diamonds) and inorganic (circles) hydroponics solution, and to MPM solution sterilized by filtration (triangles) and autoclaving (squares).

Next, we investigated the microbiota in the MPM solution without the cultivation of plants. We treated samples of the MPM solution by filtration and autoclaving to sterilize the solution and added *F. oxysporum* strain H111-dsRed to the resulting samples. Densities of *F. oxysporum* increased greatly in both the sterilized samples and the inorganic hydroponics solution (Fig. 4). The increase leveled off by 3 days after the experiment started. On the other hand, the density of the fungal pathogen did not increase in the untreated MPM solution and fluctuated slightly during the experimental period (Fig. 4). These results demonstrated that microbial exudates in microenvironment, and not copious amount of water-soluble or heat-stable substances secreted by the MPM microbes, were likely to be the main factor that controls *F. oxysporum* activity.

### Microbial density required to maintain disease suppression

**Figure 5:**
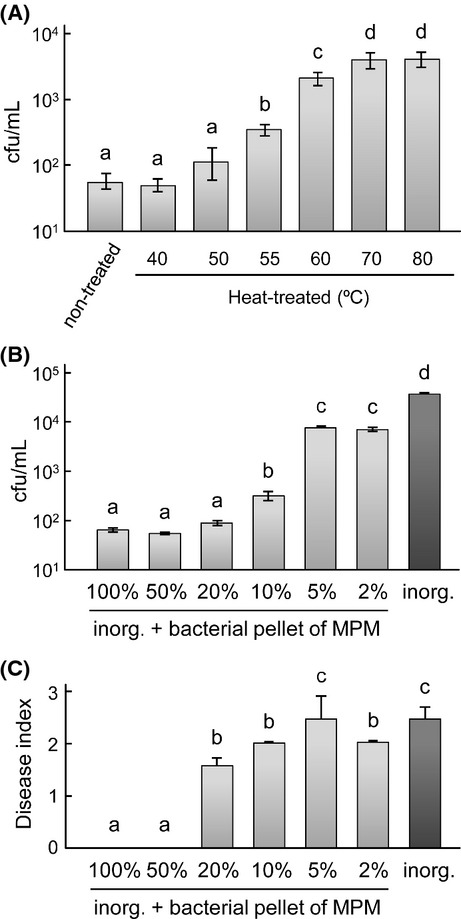
Microbial density required to maintain the ability to suppress Fusarium wilt disease. Microbiological factors affecting this suppression in the multiple parallel mineralization (MPM) system were evaluated by pasteurization or a suppression transfer test in which the MPM solution was diluted. *Fusarium oxysporum* f. sp. *lactucae* strain H111-dsRed was added after (A) pasteurization of the MPM solution at 40–80°C for 30 min or (B) dilution of the microbial pellet collected by centrifugation of the MPM solutions. The inoculated solutions were cultured at 25°C for 7 days, and were examined using Komada's selective medium supplemented with hygromycin B. (C) Suppression of Fusarium wilt disease of lettuce was maintained using the diluted microbial pellet. The disease severity was determined on a scale of 0–3, in which 0 indicates no disease, 1 indicates wilting leaves, 2 indicates a wilting plant, and 3 indicates a dead plant, and symptoms were recorded until 10 days after inoculation. The disease rating value represents the mean (±SD) for 12 seedlings from each treatment replicate and was recorded until 10 days after inoculation. Bars labeled with different letters were significantly different (*P* < 0.05, Tukey–Kramer multiple-comparison test).

We examined the growth of *F. oxysporum* in the MPM solution using several heat treatments (40–80°C). We added *F. oxysporum* strain H111-dsRed to the heat-treated solution without the cultivation of plants. The suppression of pathogen growth was retained by the MPM solution after heat treatment at temperatures of up to 50°C. However, treatment at temperatures greater than or equal to 60°C completely eliminated the solution's suppressive ability (Fig. 5A) and resulted in an increase in the pathogen's growth rate to a level similar to that in the untreated inorganic hydroponics. These results showed that the microbiota that suppressed the growth of the fungal pathogen was temperature sensitive and that the microbial community structure changed as a result of the heat shock.

We investigated whether the microbial density was responsible for the suppression of *F. oxysporum*. We used a prepared MPM solution that was centrifuged, then diluted the pellet (2–50% w/w) and resuspended it in the inorganic hydroponics solution. *Fusarium oxysporum* strain H111-dsRed was added to the prepared solution without the cultivation of plants. The microbial impact on *F. oxysporum* growth decreased as the microbial population was progressively reduced by the dilution (Fig. 5B). The growth of the inoculated *F. oxysporum* was suppressed to the same level as in the undiluted solution when at least 20% of the original amount of microbes was present. However, the pathogen growth was not inhibited at 5% or less of the original concentration, resulting in increases in the fungal cell density to ∼1 × 10^4^ CFU mL^−1^. There was a noticeable but clearly and significantly decreased suppression at 10% of the original concentration. These results help to quantify the importance of the microbiota for biocontrol in terms of their ability to suppress the pathogen's growth.

We also performed an inoculation test on lettuce plants using *F. oxysporum* strain H111-dsRed in the inorganic hydroponics solution with the diluted microbial pellets. Disease suppression decreased as the dilution of the solution increased, with nearly complete suppression at a concentration of 50% of the original, but a large reduction in the suppressive ability at 20% and little or no reduction at 10% or less (Fig. 5C). These results show that the disease suppression provided by the MPM solution depended on the microbial density.

### Morphological characterization of F. oxysporum in the rhizosphere microbial community

**Figure 6:**
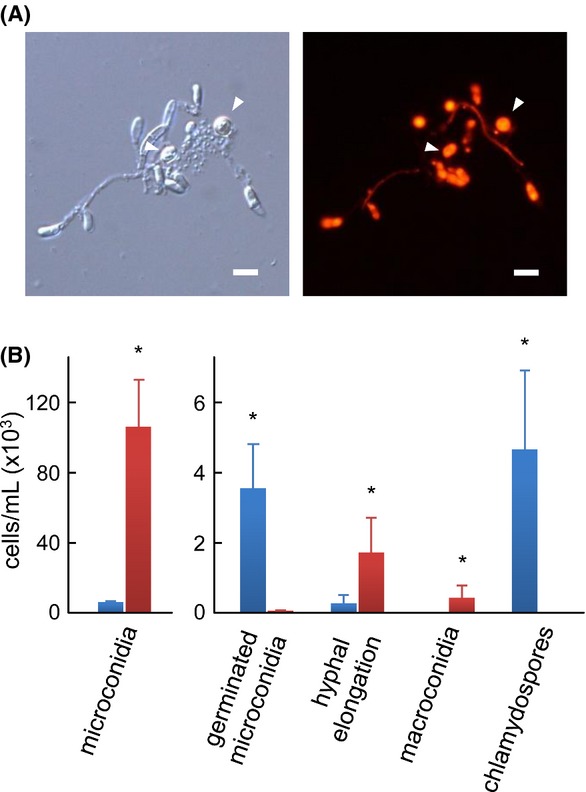
Control of *Fusarium oxysporum* morphology in the multiple parallel mineralization (MPM) solution. *F. oxysporum* f. sp. *lactucae* strain H111-dsRed was added to the MPM and inorganic hydroponics solutions, and cultured at 25°C for 7 days. (A) Survival of cells of the transformants in the MPM solution was observed using a light microscope. Strain H111-dsRed exhibited red fluorescence in the MPM solution. Chlamydospores (arrowheads) formed on the bacterial cells. Bars represent 10 mm. (B) Cell formation of *F. oxysporum* f. sp. *lactucae* strain H111-dsRed was assessed using the numbers of microconidia, germinated microconidia (<20 *μ*m), macroconidia, and chlamydospores and by the number of microconidia that exhibited hyphal elongation >20 *μ*m. Blue bars, MPM system; red bars, inorganic hydroponics system. Bars labeled with a star were significantly different between the two solutions (*P* < 0.05, Student's *t*-test).

We compared the formation of conidia and the status of the inoculated microconidia of *F. oxysporum* strain H111-dsRed between the MPM and the inorganic hydroponics solutions for 7 days after incubation. The fungal cells in the MPM solution exhibited dsRed fluorescence, indicating that the *F. oxysporum* inhabiting the MPM solution was alive (Fig. 6A). *Fusarium oxysporum* cells in the MPM solution formed microconidia that germinated and chlamydospores, whereas those from the inorganic hydroponics solution were likely to produce microconidia and macroconidia (Fig. 6B). The growth of fungal cells through the production of microconidia and macroconidia resulted in an increase to ∼1 × 10^5^ CFU mL^−1^ in the inorganic hydroponics solution. In contrast, the density of fungal cells in the MPM solution remained stable at ∼1 × 10^4^ CFU mL^−1^. Macroconidia production was not identified in the MPM solution (Fig. 6B). Microconidial germination occurred in the MPM solution, accounting for ∼30% of the *F. oxysporum* cells (Fig. 6B). The germinated microconidia in the MPM solution showed dramatic changes in morphology, and we identified chlamydospores with a preserved lipid body and thick walls that let the resting spore survive unfavorable conditions (data not shown). The level of chlamydospore production varied depending on the microbial density in the MPM solution. At a dilution to 10% or less of the original concentration, chlamydospore formation decreased dramatically (data not shown). These results showed that *F. oxysporum* in the MPM solution retained viable cells in an inactive state under the unfavorable MPM solution conditions. These findings indicate that microbial consortia in the MPM solution prevented *F. oxysporum* from elongation of its hyphae and from production of conidia.

## Discussion

A major objective of this study was to evaluate the suppression of fungal disease by MPM system. Our results demonstrated that the community of rhizosphere microbiota that developed in the MPM system could suppress *F. oxysporum* in both in vitro studies and in experiments performed under greenhouse conditions. Previous studies provided some evidence that the production of antimicrobial substances, control of fungal morphogenesis, and induction of host systemic resistance play key roles in soils that are capable of suppressing Fusarium wilt (Scher and Baker 1980; Alabouvette 1986; Weller et al. 2002; Haas and Défago 2005). In this study, our DGGE profiles revealed that the microbiota, including important bacteria in *Bacillus* spp., was present in the rhizosphere of the MPM system. Members of genus *Bacillus* are known to be potential biocontrol agents for *F. oxysporum* (Weller et al. 2002) and produce a wide range of antibiotics from different functional classes (Raaijmakers et al. 2002). We further tested the biocontrol abilities against *F. oxysporum* under in vitro conditions using several treatments and demonstrated that microbial survivability was strongly associated with the biocontrol efficacy, suggesting that antimicrobial substances that are diffusible, degradable, or sensitive to heat and concentration were unlikely to be the primary cause of disease suppression. We are currently investigating possible antimicrobial agents that include volatile organic compounds (Minerdi et al. 2009).

Control of *F. oxysporum* morphogenesis by rhizosphere microbes is part of the mechanism responsible for disease suppression by the MPM system. Microbial interactions with *F. oxysporum* in the microenvironment stimulated chlamydospore formation and inhibited reproduction of *F. oxysporum*. The chlamydospores of *F. oxysporum* can form under unfavorable environmental conditions, such as at low temperature, and these resting bodies act as primary inocula in subsequent soil-borne infections, whereas the microconidia and macroconidia formed by *F. oxysporum* are important in secondary infection (Nelson 1981; Couteaudier and Alabouvette 1990). In this study, *F. oxysporum* survived in the MPM system by forming chlamydospores when they were exposed to the rhizosphere microbiota, even though there were few chlamydospores 3 days after inoculation (Fig. S5). The surviving fungal cells did not cause Fusarium wilt disease in the MPM system, but retained their pathogenicity when they were reisolated from the solution. The *F. oxysporum* cells in the MPM solution had a higher rate of microconidia germination, but a lower hyphal elongation rate than those in the inorganic hydroponics solution. In this species, hyphal elongation is necessary for the production of microconidia and macroconidia (Nelson 1981). Thus, the decreased hyphal elongation in the MPM solution may prevent an increase in the numbers of macroconidia and microconidia, resulting in an unchanged *F. oxysporum* cell density. Ford et al. (1970) accounted for microbial stimuli of *Fusarium solani* activity and demonstrated that the inclusion of soil microbes in the solution induced chlamydospore formation. Some bacteria that are known to be biocontrol agents adhere to the hyphae of *F. oxysporum* (Elvers et al. 2001) and alter its hyphal morphology (Bolwerk et al. 2003). Minerdi et al. (2008) reported that microbial symbionts silence the virulence of *F. oxysporum* and that changes in cell morphogenesis by *F. oxysporum* underlie this suppression. These findings suggest that the rhizosphere microbial consortia that become established in the MPM system are unsuitable for the growth of *F. oxysporum* and negatively affect its pathogenic ability as a result of changes in its morphogenesis.

Bacterial interactions with plant roots can lead to plant resistance to *F. oxysporum*. Induced systemic resistance (ISR) against this pathogen can be elicited by some strains in the genera *Bacillus*, *Pseudomonas*, *Serratia*, and *Achromobacter* (Van Peer et al. 1991; Someya et al. 2000; Lugtenberg and Kamilova 2009). In this study, we demonstrated that one or more potential biocontrol agents in genus *Bacillus* existed in the MPM system. Although ISR may be one of the causal factors for the suppression of root-borne diseases in the MPM system, there is conflicting evidence as to whether ISR plays a major role in disease suppression. Our results indicated that tomato plants grown in the MPM system were still susceptible to diseases of the aerial parts of the plant, including airborne pathogens such as powdery mildew (*Oidium neolycopersici*), leaf mold (*Cladosporium fulvum*), or gray mold (*Botryotinia fuckeliana*) during cultivation in the greenhouse (M. Shinohara and K. Fujiwara, unpubl. data). Because *F. oxysporum* exhibited morphological changes that allowed it to survive in the rhizosphere microbiota, but with reduced infectious ability, the disease suppression we observed in the MPM system is most likely to result from microbiological interactions rather than from ISR.

Fusarium wilt disease suppression in the MPM system can be ascribed to microbiota that controls the morphological changes in *F. oxysporum*. However, little is known about the development of disease suppression in the MPM system. In this study, the development of functional abilities of the MPM solution capable of influencing Fusarium wilt disease suppression and altering fungal morphogenesis was not found when the pathogen was inoculated within 4 days after transplanting. We hypothesize that the absence of biocontrol abilities during this early stage results from incomplete biofilm formation in the rhizosphere that prevents the microbiota from conferring their biocontrol ability due to insufficient microbe–microbe or plant–microbe interactions. Our previous study provided evidence that a thick biofilm begins to form about 1 day after transplanting, followed by the development of a thinner biofilm as a result of changes in the rhizosphere structures involving the roots, and that stable biofilm formation occurs around 4 days after transplanting (Fujiwara et al. 2012). The present results agree with that previous finding. Because it is well known that biofilm formation does not simply cause the development of disease suppression, we further predict that certain as-yet-unidentified causal factors influence the stability of the rhizosphere microbiota and their biocontrol abilities. Past investigations suggest that microbial communication physically and functionally contributes to the establishment of microbiota that is associated with biocontrol activities (Raaijmakers and Mazzola 2012; Chen et al. 2013). In future research, we will need to elucidate the causal factors responsible for the development of biocontrol effects that lead to the suppression of root-borne diseases.

## Supporting Information

**Figure S1.** Microscopic observation of biofilms adhering to the root surface in the MPM system. Tomato roots were grown in the MPM system for 4 days after transplanting. Seedlings were cultivated in a greenhouse at a constant day and night temperature of 30°C. Rhizosphere microbes inhabiting the root surface were imaged using (A) a digital microscope and (B) a scanning electron microscope. The regions bounded by the red dotted lines are magnified in Figure 1C.

**Figure S2.** Phylogenetic tree of the rhizosphere microorganisms detected in the biofilms from the MPM system. Based on the nucleotide sequences of the 16S ribosomal DNA, the phylogenetic relationships among 29 isolates were analyzed using the neighbor-joining method provided by version 2.10 of the DNASIS Pro software (Hitachi Software Engineering Co., Tokyo, Japan). The stability of groups was assessed using 1000 bootstrap replications of the data sets. Letters in parentheses from each phylogenetic taxon represents the band labels in the DGGE banding profiles for the bacterial 16S rDNA genes obtained from the rhizosphere biofilms (Fig. 2).

**Figure S3.** UPGMA dendrogram of the rhizosphere microbial community constructed using the Bray–Curtis index. Clustering analysis was performed using an unweighted pair group method with arithmetic mean (UPGMA) based on Bray–Curtis indices to evaluate the percentage similarity among the samples using the vegdist function of the vegan package for the R statistical software (Cleary et al. 2012). For this analysis, each DGGE band was assumed to be an OTU and assigned to a bacterial phylum. A permutation test was performed based on the values in the Bray–Curtis distance matrix using the adonis function provided by the vegan package (Oksanen 2011). The number of permutations was set at 999; all other parameters used the default values set in the function. The scale shows the Bray–Curtis similarity value. Sample names correspond to the band lanes shown in Figure 2

**Figure S4.** The MPM system suppressed the Fusarium wilt disease. (A) Boston lettuce (*Lactuca sativa* L. cv. Saradana) was grown in the MPM and inorganic hydroponics systems in a greenhouse. Plants were inoculated with *Fusarium oxysporum* f. sp. *lactucae* strain H111 (1 × 10^4^ cells mL^−1^) 4 days after transplanting and grown for 1 month. The image shows the results 30 days after inoculation. (B) Tomato plants (*Solanum lycopersicum* L. cv. Ponderosa) were inoculated with *F. oxysporum* f. sp. *radicis-lycopersici* strain LS89-1-1 (1 × 10^4^ cells mL^−1^) 4 days after transplanting. The image shows the results 10 days after inoculation. Disease severity in the tomato plants is shown in Table S2.

**Figure S5.** Observations of *Fusarium oxysporum* morphology in the MPM solution. (A) *Fusarium oxysporum* f. sp. *lactucae* strain H111-dsRed was added to the MPM and inorganic hydroponics solutions (1 × 10^4^ cells mL^−1^), and the inoculated solutions were cultured at 25°C for 7 days. H111-dsRed produced typical microconidia, germinated microconidia, elongating hyphae, and macroconidia. Bars represent 10 *μ*m. (B) Cell formation by *F. oxysporum* f. sp. *lactucae* strain H111-dsRed was assessed 1 and 3 days after inoculation based on the numbers of microconidia, germinated microconidia, macroconidia, and chlamydospores and by the number of hyphae that elongated (>20 *μ*m). Blue bars, MPM system; red bars, inorganic hydroponics system. Bars labeled with a star were significantly different (*P* < 0.05, Student's *t*-test). (C) Bacterial adhesion on chlamydospores of *F. oxysporum* f. sp. *lactucae* strain H111-dsRed. Survival of the transformants in the MPM solution was observed with a light microscope. Chlamydospores (arrowheads) formed in contact with the bacterial cells. Bars represent 10 mm.

**Data S1.** Supplemental experimental procedures.

**Table S1.** Primers used in this study.

**Table S2.** Timing and severity of symptom development in Boston lettuce and tomato plants inoculated with *Fusarium oxysporum* f. sp. *lactucae* and *F. oxysporum* f. sp. *radicis-lycopersici* in the MPM system.
