# Supplementary figures and images for: The rhizosphere microbial community in a multiple parallel mineralization system suppresses the pathogenic fungus Fusarium oxysporum
Source: Microbiologyopen. 2013 Nov 8;2(6):997–1009. doi: 10.1002/mbo3.140 (PMC3892345; doi:10.1002/mbo3.140)

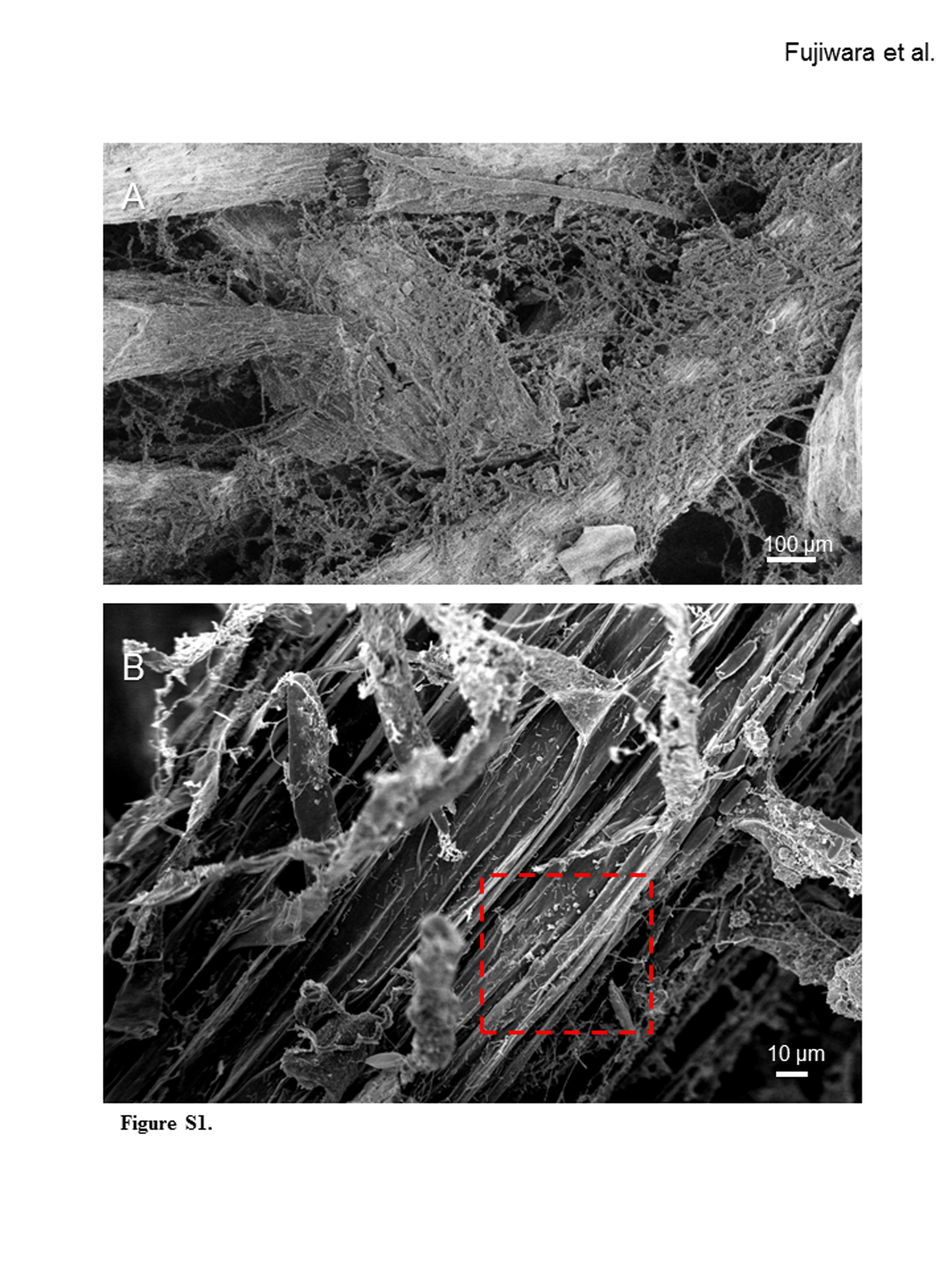

Supplement: Supplementary file 1 [file mbo30002-0997-SD1.tif]

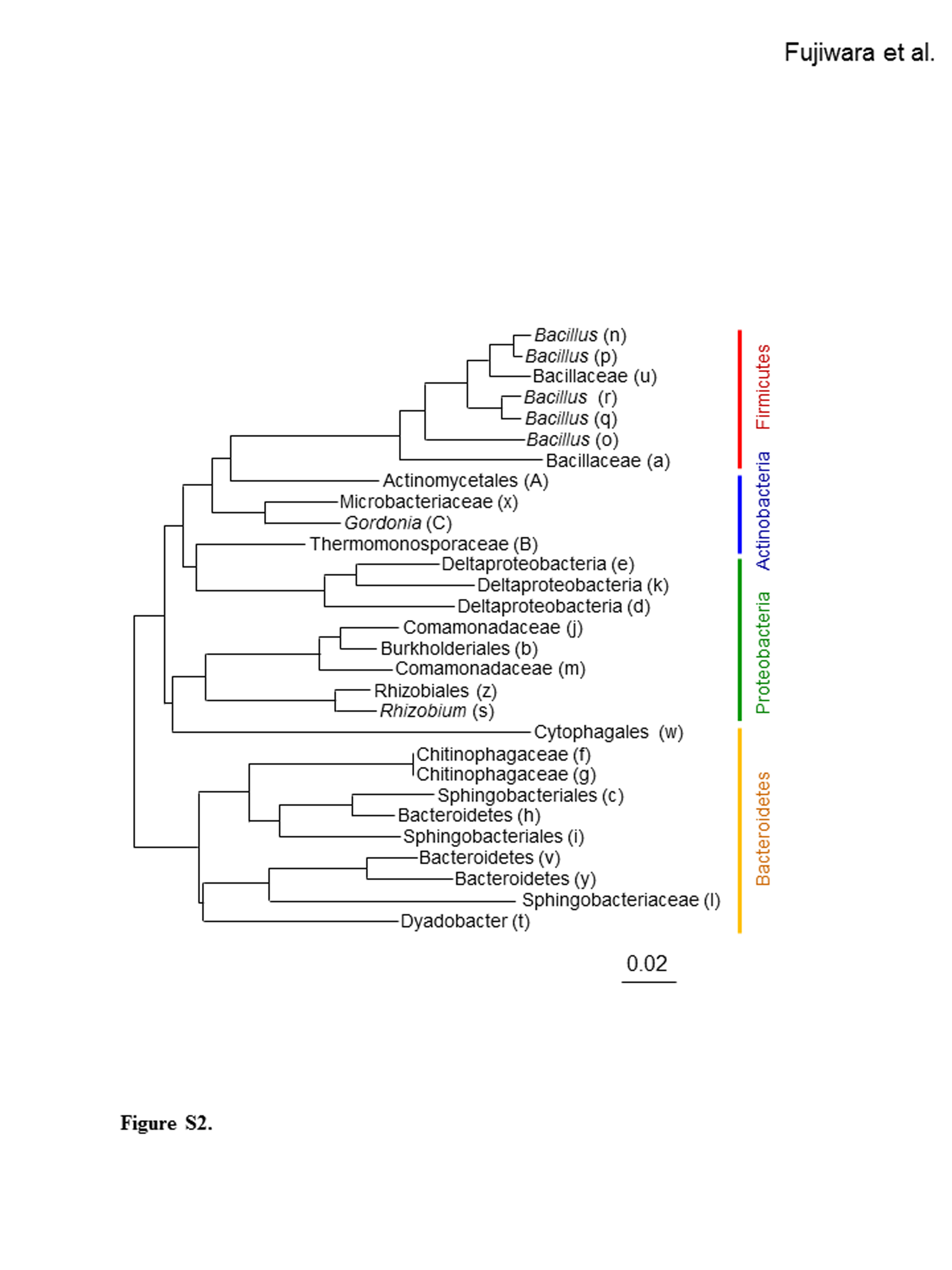

Supplement: Supplementary file 2 [file mbo30002-0997-SD2.tif]

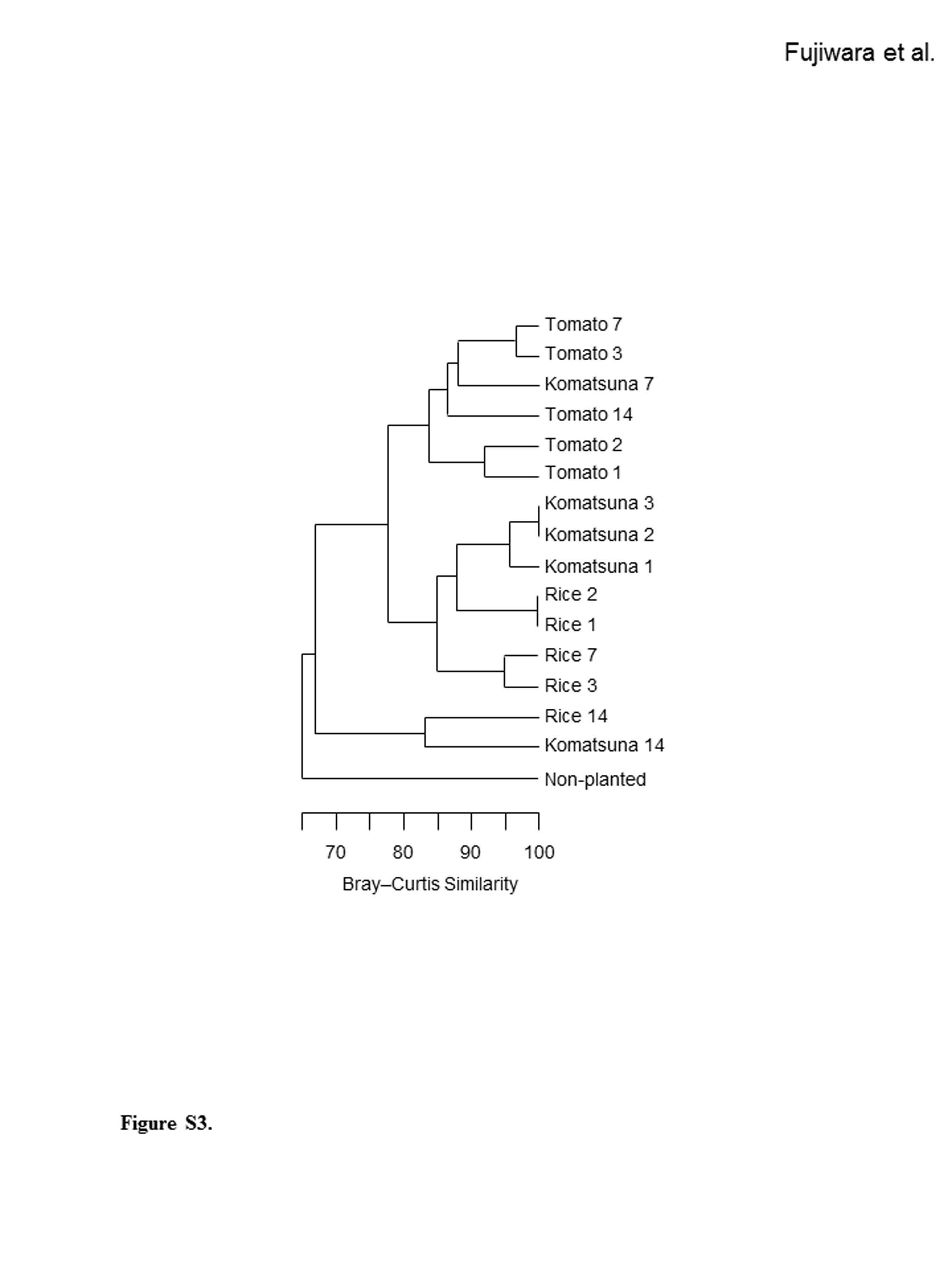

Supplement: Supplementary file 3 [file mbo30002-0997-SD3.tif]

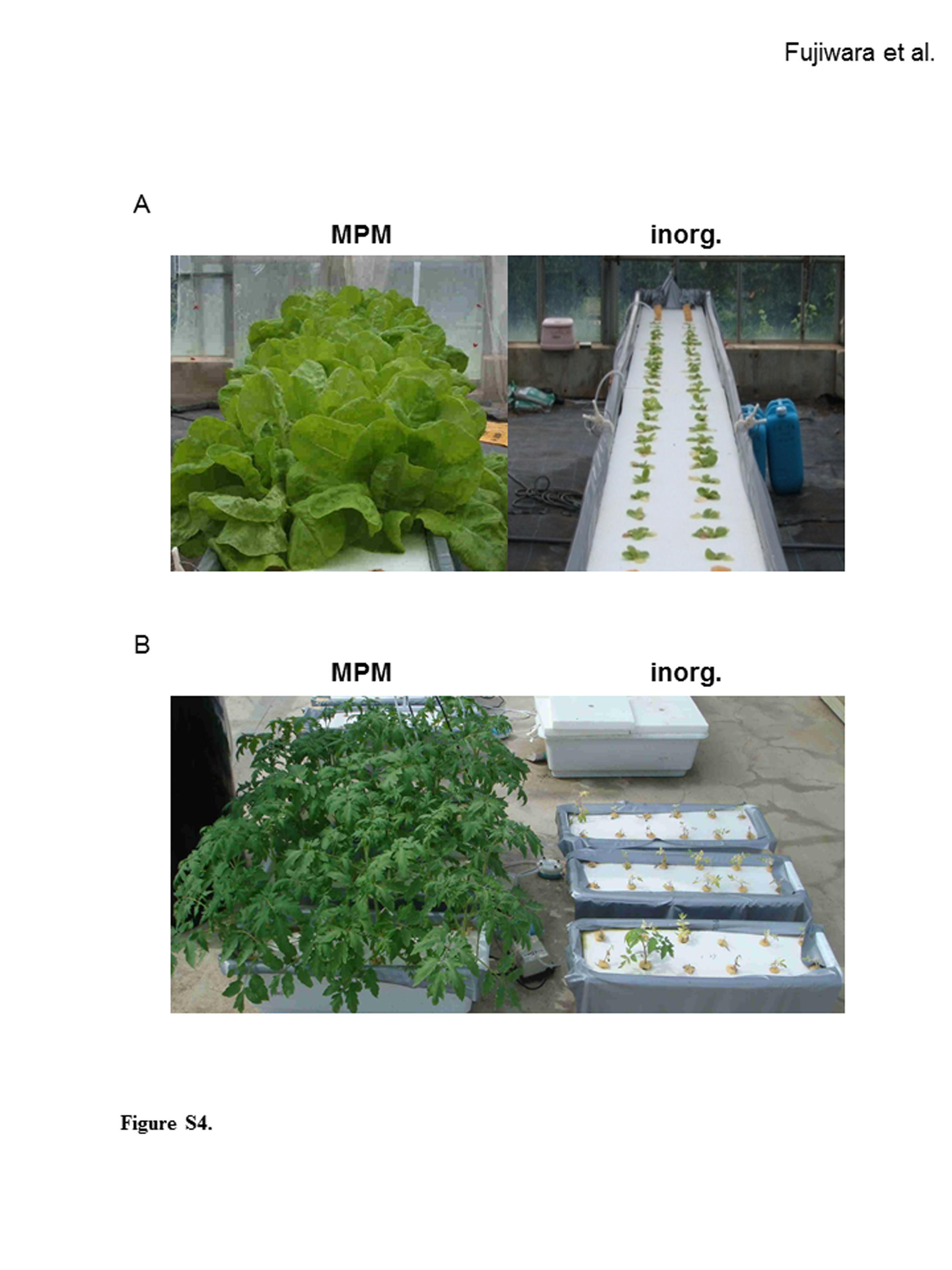

Supplement: Supplementary file 4 [file mbo30002-0997-SD4.tif]

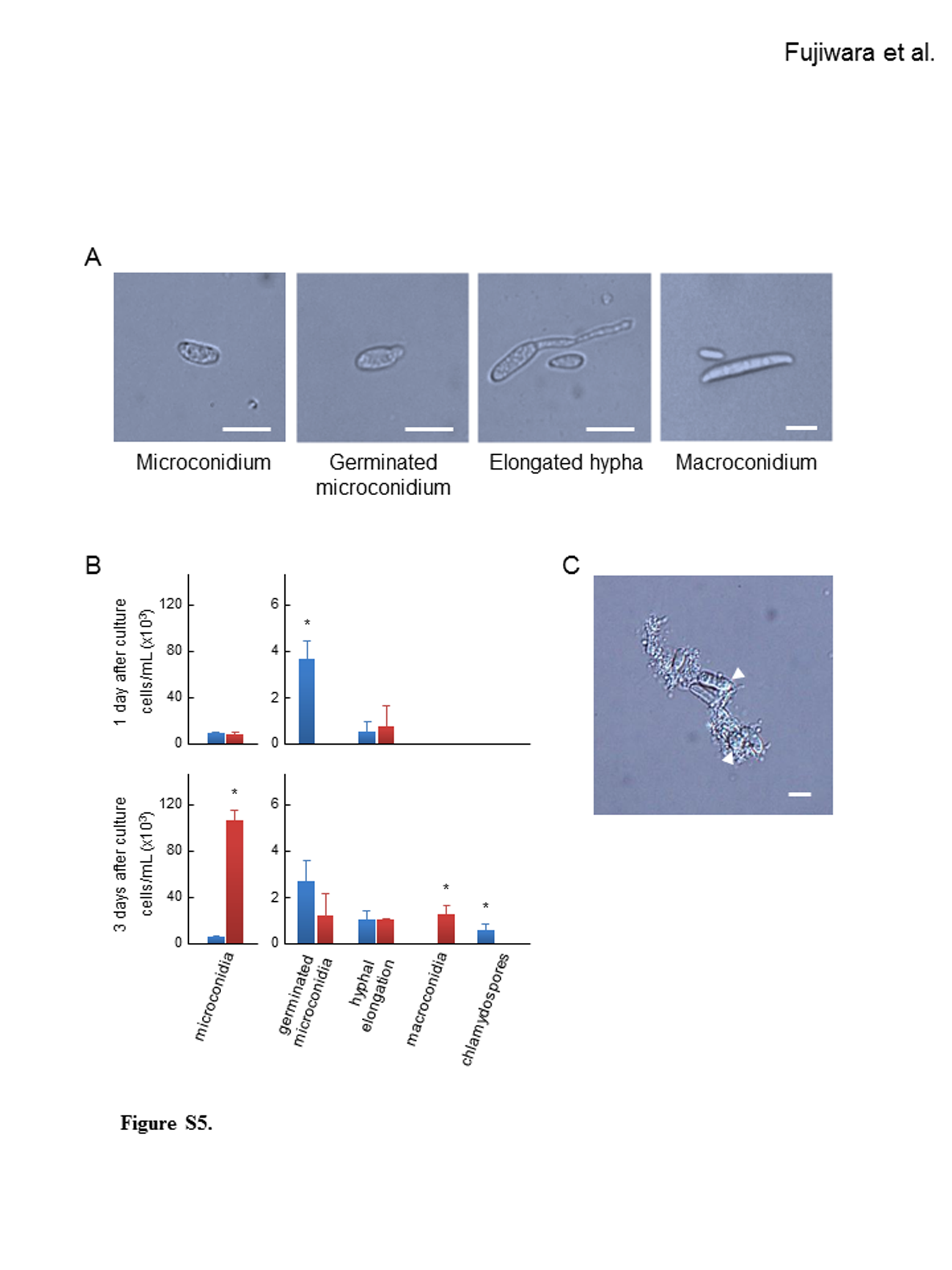

Supplement: Supplementary file 5 [file mbo30002-0997-SD5.tif]
