# Supplementary material for: The rhizosphere microbial community in a multiple parallel mineralization system suppresses the pathogenic fungus Fusarium oxysporum
Source: Microbiologyopen. 2013 Nov 8;2(6):997–1009. doi: 10.1002/mbo3.140 (PMC3892345; doi:10.1002/mbo3.140)
Supplement: Supplementary file 6 [file mbo30002-0997-SD6.doc]

**Supporting Information**

**Supplemental experimental procedures**

*Multiple parallel mineralization for production of the MPM solution*

The MPM solution, which is the hydroponics solution used in the MPM system, was prepared by a procedure that we have called “multiple parallel mineralization” (Shinohara et al. 2011). This procedure aims at culturing a community of microorganisms in water that are capable of mineralizing organic nitrogen into nitrate nitrogen. Creation of this solution requires only four steps: addition of soil, addition of organic substances, addition of oystershell lime to the water, and aeration of the water. First, we prepared 15 L of water containing 150 g of nursery soil as a microbial inoculum (Nae-ichiban; Sumirin Agro-Products, Aichi, Japan). The soils we used were bagged in a nonwoven fabric bag (“Toshanse”, Seiketsu network, Osaka, Japan) before there were added. We removed the soils added to the water when more than 5 mg L^–1^ of nitrate was detected. Second, we added 28 g of corn steep liquor (CSL; (Nature Aid; Sakata Seed, Yokohama, Japan), which contains 30 mg N g^–1^, to the water as a source of organic nitrogen for the microbes. In this study, we added 4 g of CSL daily for 7 days. Next, we added 150 g of oystershell lime (Ryujyoseruka; Urabe Sangyo, Hiroshima, Japan) to the water as a supplement to provide micronutrients. The oystershell lime was bagged in a nonwoven fabric bag before it was added to the solution. Finally, we aerated the solution using an aeration pump and held the solution at room temperature for more than 2 weeks. We maintained the aeration until nitrate levels reached at least 200 mg L^–1^ and the concentration stabilized. We regarded this condition to represent complete mineralization of the CSL. The nitrate concentration was measured daily using an RQ-Flex Plus Analyzer (Merck, Frankfurt, Germany). More details on the procedure, such as the types of soil and organic substances available for the procedure, were described in our previous report (Shinohara et al. 2011).

**Table S1.** Primers used in this study ^a^.

| Name | Sequence (5' → 3') |
| --- | --- |
| GC-341f | CCT ACG GGA GGC AGC AG |
| GC-534r | ATT ACC GCG GCT GCT GG |
| Ptef-RFP | CCT CGG AGG AGG CCA TGT TTG ACG GTG ATG TAT GGA |
| Tgla-RFP | CCA CCT GTT CCT GTA GAC AAT CAA TCC ATT TCG CTA |
| RFP-F | *ATG* GCC TCC TCC GAG GAC GT |
| RFP-R | *CTA* CAG GAA CAG GTG GTG GC |

^a^ The primers have 16 bases (underlined) that are identical to the sequences at the ends of the dsRed gene. The initiation and termination codons are indicated in italics.

**Table S2.** Timing and severity of symptom development in Boston lettuce and tomato plants inoculated with *F. oxysporum* ff. spp. *lactucae* and *radicis-lycopersici* in the MPM system^a^.

| Treatment ^b^ | Disease rating at 5 to 10 days after inoculation | | | | | |
| --- | --- | --- | --- | --- | --- | --- |
|  | 5 | 6 | 7 | 8 | 9 | 10 |
| MPM | 0.0 (± 0.0) | 0.0 (± 0.0) | 0.0 (± 0.0) | 0.0 (± 0.0) | 0.0 (± 0.0) | 0.0 (± 0.0) |
| MPM inoculated  with *F. o*. *l.* | 0.0 (± 0.0) | 0.0 (± 0.0) | 0.0 (± 0.0) | 0.0 (± 0.0) | 0.0 (± 0.0) | 0.0 (± 0.0) |
| inorg. inoculated  with *F. o*. *l.* | 0.0 (± 0.0) | 1.4 (± 0.3) | 1.8 (± 0.2) | 2.2 (± 0.3) | 2.3 (± 0.2) | 2.4 (± 0.3) |
| MPM inoculated  with *F. o*. *r.* | 0.0 (± 0.0) | 0.0 (± 0.0) | 0.0 (± 0.0) | 0.0 (± 0.0) | 0.0 (± 0.0) | 0.0 (± 0.0) |
| inorg. inoculated  with *F. o*. *r.* | 0.0 (± 0.0) | 0.7 (± 0.2) | 1.2 (± 0.2) | 1.4 (± 0.3) | 1.75 (± 0.3) | 2.3(± 0.4) |

^a^ Disease rating: For tomato, 0, no symptoms; 1, yellowing; 2, wilting; 3, death. For lettuce, 0, no symptoms; 1, wilting leaves; 2, a wilting plant; 3, death. Values are the means (±S.D.) of the disease ratings from 12 lettuce (*F. o*. *l.*) and 36 tomato plants (*F. o*. *r.*) in each treatment replicate. The symptoms 10 days after inoculation are shown in supplemental Figure S4.

^b^ Plants cultivated in the MPM and inorganic (inorg.) hydroponics systems for 4 days after transplanting were inoculated with 1×10^4^ cells mL^–1^ of *F. oxysporum* f. sp. *lactucae* (*F. o*. *l.*) or f. sp. *radicis-lycopersici* (*F. o*. *r.*).
